# Induction chemotherapy plus concurrent chemoradiotherapy versus induction chemotherapy plus volumetric modulated arc therapy alone in the treatment of stage II-IVB nasopharyngeal carcinoma patients: a retrospective controlled study

**DOI:** 10.1186/s13014-018-1092-0

**Published:** 2018-08-13

**Authors:** Linger Liu, Zhenghua Fei, Mengfeng Chen, Lihao Zhao, Huafang Su, Dianna Gu, Baochai Lin, Xiaona Cai, Lihuai Lu, Mengdan Gao, Xuxue Ye, Xiance Jin, Congying Xie

**Affiliations:** 1 Department of Radiation and Medical Oncology, The First Affiliated Hospital of Wenzhou Medical University, No.2 Fuxue Lane, Wenzhou, 325000 China; 2 Department of Oncology Medicine, Yueqing 3rd People’s Hospital, Wenzhou, 325600 China

**Keywords:** Nasopharyngeal carcinoma, Concurrent chemotherapy, Volumetric modulated arc therapy, Toxicity, Survival outcome

## Abstract

**Background:**

In the era of intensity-modulated radiotherapy (IMRT), the role of additional concurrent chemotherapy (CC) to radiotherapy (RT) after induction chemotherapy (IC) compared to IC followed by RT alone remains unclear for stage II-IVB nasopharyngeal carcinoma (NPC) patients. The aim of this study was to evaluate the efficacy and toxicities of IC/RT and IC/CCRT in the treatment of NPC with volumetric modulated arc therapy (VMAT).

**Methods:**

From January 2012 to March 2016, a total of 217 NPC patients were retrospectively assessed. Of the 217 patients, 139 patients received IC followed by VMAT alone and 78 patients received IC plus CCRT. Overall survival (OS), progression-free survival (PFS) and toxicities were assessed.

**Results:**

The 5-year OS, PFS rates were 57.5%, 41.8% and 47.8%, 38.4% for the IC/RT and IC/CCRT arms, respectively, without significant difference in survival between the two groups (both *p* > 0.05). Multivariate analysis indicated that treatment modality (IC/RT vs. IC/CCRT) was not an independent prognostic factor for OS or PFS. Grade 3–4 leukopenia/neutropenia (3.60% vs. 20.51%, *p* < 0.001), gastrointestinal disorder (nausea/vomiting/diarrhea, 2.16% vs. 41.03%, *p* < 0.001), mucositis (29.50% vs. 47.44%, *p* = 0.01) and xerostomia (34.53% vs. 48.72%, *p* = 0.04) were more frequent in the IC/ CCRT arm than in the IC/RT arm during VMAT.

**Conclusions:**

No significant difference in OS and PFS was observed between IC plus VMAT alone and IC/CCRT in the treatment of stage II-IVB NPC patients, however, more side effects were observed in the IC/CCRT arm.

## Background

Nasopharyngeal carcinoma (NPC) is endemic in Southern China, with an annual incidence of 15–50 cases per 100,000 [1]. Due to its high sensitivity to chemotherapy and radiotherapy, combined chemotherapy and radiotherapy has been the standard treatment modality for stage II-IVB NPC as recommended in the NCCN guidelines [2].

The strategy of induction chemotherapy (IC) followed by concurrent chemoradiotherapy (CCRT) is widely applied for NPC patients in China. Studies demonstrated that IC followed by CCRT significantly improved survival in NPC [3–5]. Promising results of IC/CCRT in NPC with 3-year progression-free survival (PFS) and overall survival (OS) rates of 88.2 and 94.1% [6], and 5-year OS rate of 78% [7], respectively were also reported. However, high incidences of grade 3/4 adverse events were observed during concurrent chemotherapy (CC) and many patients failed to complete the course of CC due to drug-related toxicities in clinical practice [8–11]. On the other hand, similar survival outcomes between IC/RT and IC/CCRT arms had been demonstrated by some trials [12, 13]. Encouraging results of IC followed by RT alone with benefit in response rate (RR) [14] and improvement in disease-free survival (DFS) [14, 15] had also been reported. To our knowledge, it remains controversial whether the addition of CC to RT after IC (IC/CCRT) improves the efficacy of treatment compared with IC/RT.

Intensity modality radiotherapy (IMRT), especially new IMRT delivery modality volumetric modulated arc therapy (VMAT) has widely replaced the conventional radiotherapy due to its better dose painting ability and efficacy in the treatment of NPC [16–18]. Therefore, we assume that IC plus IMRT alone might be a feasible option with reduced toxicities in the treatment of NPC. What is more, most previous studies were based on conventional radiotherapy. Few studies compared the outcomes between IC/RT and IC/ CCRT arms with VMAT in the treatment of NPC. Therefore, the aim of this study was to evaluate the efficacy and toxicities of IC/RT and IC/CCRT with VMAT in the treatment of stage II-IVB NPC patients.

## Methods

### Patients

**Fig. 1:**
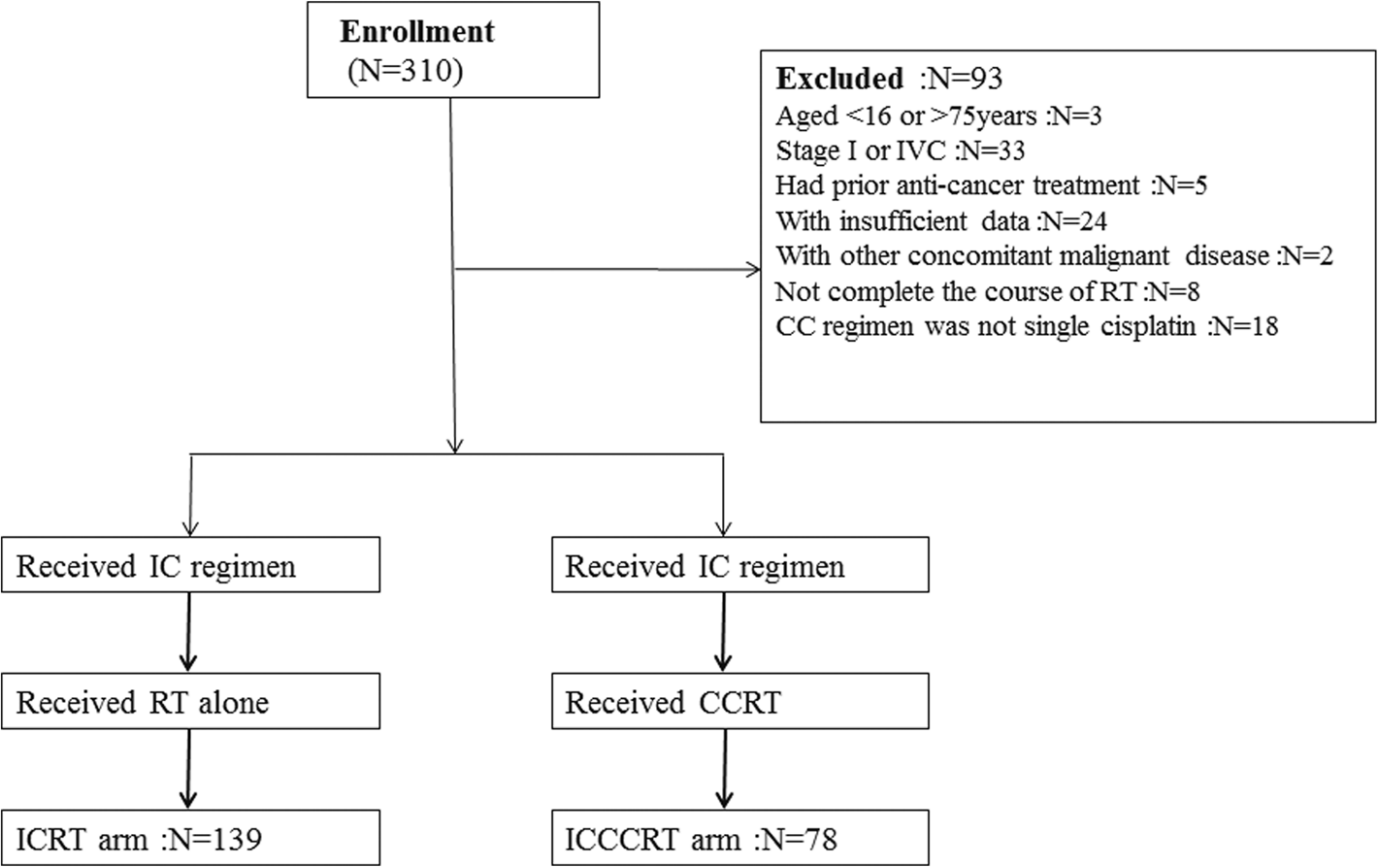
Inclusion and exclusion criteria flow diagram of all patients

We retrospectively reviewed all NPC patients underwent chemotherapy and VMAT from January 2012 to March 2016 in author’s institution. The flow diagram for study design of this study was shown in Fig. 1. Patients were divided into two arms: IC/RT and IC/CCRT. Patients in these two groups were matched for seven characteristics: age (≤ 54 years vs. > 54 years), sex (male vs. female), T category (T1 vs. T2 vs. T3 vs. T4), N category (N0 vs. N1 vs. N2 vs. N3), clinical stage (II vs. III vs. IVA vs. IVB), IC regimen (cisplatin plus fluorouracil (FP) vs. docetaxel/paclitaxel plus cisplatin (TP) vs. other) and number of IC cycles (≤ 2 vs. > 2 cycles).

Inclusion criteria for this study were as follows: (i) New histologically confirmed NPC; (ii) Stage II-IVB at diagnosis (the 7th edition of American Joint Committee on Cancer) [2]; (iii) Aged 16 to 75 years-old; (iv) Adequate liver, renal and hematologic function (absolute neutrophil ≥1.5 × 10^9^/L or platelet count ≥100 × 10^9^/L); (v) Karnofsky performance score ≥ 70; (vi) No previous malignancy or other concomitant malignant disease; (vii) No prior anti-cancer treatment. The exclusion criteria were: (i) Age < 16 or > 75 years-old; (ii) Stage I or stage IVC NPC; (iii) Pregnancy; (iv) Patients with insufficient data. This study was approved by the Institutional Review Board and performed at author’s institution.

### Treatment

All patients underwent VMAT with simultaneous integrated boost (SIB) technique 2 weeks after IC. Target and normal tissue delineations have been reported in our previous study and generalized here only briefly. Gross tumor volume (GTV) was delineated as the mass shown in the enhanced CT images and/or MRI images, including the nasopharyngeal tumor, retropharyngeal lymphadenopathy, and enlarged neck nodes. For the GTV of the primary tumor, involved retropharyngeal lymph nodes and intracavity lesions were delineated according to the post-IC volume, whereas involved tissues (eg, pterygopalatine fossa) were delineated according to the pre-IC volume of the primary lesion as shown by MRI. Post- IC volumes were used to delineate the rest involved lymph nodes. Clinical target volume (CTV) was defined as the GTV plus a margin of potential microscopic spread. Planning target volume (PTV) was created by adding a 3 mm margin to the CTV to account for setup variability [19].

Prescription doses were 70 Gy and 56 Gy for GTV and CTV in 28 fractions, respectively. OARs consisting of the brainstem, spinal cord, left and right parotids were included for optimization. Dual arc VMAT plans were generated on Philips Pinnacle^3^ treatment planning system (TPS) (clinical version 9.2; Philips, Fichburg, WI, USA). Optimization parameters and process have been reported in our previous study. Briefly, the first arc rotates clockwise with a start angle of 181° and a stop angle of 180°, and the second arc rotates counterclockwise from 180° to 181°. During the optimization, leaf motion of 0.46 cm/deg. and a final arc space degree of 4 were employed [16].

Induction chemotherapy was composed of TP, FP, and gemcitabine plus cisplatin. All regimens were given every 3 weeks. The concurrent chemotherapy regimen was 80–100 mg/m^2^ cisplatin on day 1 every 3 weeks for 2–3 courses.

### Assessments and follow-up

The efficacy of treatment was evaluated according to the Response Evaluation Criteria in Solid Tumors (RECIST) version 1.1 at two time points: at the end of RT, and 3 months after RT. Chemotherapy-related toxicities were evaluated according to the Common Terminology Criteria for Adverse Events (CTCAE, version 3.0). RT-induced toxicities were graded according to the Acute and Late Radiation Morbidity Scoring Criteria of the Radiation Therapy Oncology Group. Patients were evaluated weekly during treatment and every 3 months for the first 3 years, every 6 months in the fourth and fifth years and annually thereafter until death. Each follow-up included physical examination, blood count measurement, liver function test, renal function test, neurologic examination, endoscopic biopsy, computed tomography (CT) scan and magnetic resonance imaging (MRI). Additional examinations were performed to evaluate distant metastasis or local relapse when indicated.

### Endpoints and statistical analysis

The study’s end points were OS (time from treatment to death of any cause or last follow-up), PFS (interval between the initiation of treatment to first disease progression, including local recurrence, distant metastasis or death due to NPC), and treatment-related toxicities. If the complete survival time was impossible to obtain or the disease did not progress, the patient’s status was assumed as the last known survival and/or contact date. Characteristics of patients and treatment-related adverse effects were compared using Pearson chi-square or Fisher’s exact tests. Survival rates and univariate analyses were estimated by Kaplan-Meier method and compared with log-rank test. Multivariate analyses were calculated using Cox proportional hazard model. All statistical analyses were performed using SPSS (version 22.0, SPSS Inc., Chicago, IL). Two-tailed *P*-values < 0.05 were considered statistically significant.

## Results

### Baseline characteristics

Ultimately, 217 patients with stage II-IVB NPC who had been treated with IC/RT or IC/CCRT were enrolled in this study, where 139 patients received IC followed by VMAT alone and 78 patients received IC plus CCRT, respectively. Baseline characteristics of patients were presented in Table 1 with a median age of 54 years old (range: 16–75 years). There were 166/217 (76.50%) male and 51/217 (23.50%) female patients, respectively, where patients in stage II, III, IVA and IVB NPC were 64/217 (29.49%), 113/217 (52.07%), 26/217 (11.98%) and 14/217 (6.45%), respectively.

**Table 1:** Characteristics of nasopharyngeal carcinoma patients with stage II-IVB

	ICRT arm (%)	ICCCRT arm (%)	*P*-value
Age (years)			0.55
≤ 54 years	69 (49.64)	42 (53.85)	
> 54 years	70 (50.36)	36 (46.15)	
Sex			0.66
Male	105 (75.54)	61 (78.21)	
Female	34 (24.46)	17 (21.79)	
T category			0.21
T1	61 (43.88)	33 (42.31)	
T2	43 (30.94)	16 (20.51)	
T3	20 (14.39)	16 (20.51)	
T4	15 (10.79)	13 (16.67)	
N category			0.19
N0	7 (5.04)	3 (3.85)	
N1	47 (33.81)	19 (24.36)	
N2	74 (53.24)	53 (67.95)	
N3	11 (7.91)	3 (3.85)	
Clinical stage			0.13
II	46 (33.09)	18 (23.08)	
III	69 (49.64)	44 (56.41)	
IVA	13 (9.35)	13 (16.67)	
IVB	11 (7.91)	3 (3.85)	
IC regimen			0.11
FP	16 (11.51)	13 (16.67)	
TP	106 (76.26)	49 (62.82)	
other	17 (12.23)	16 (20.51)	
IC cycles			0.78
≤ 2 cycles	81 (58.27)	47 (60.26)	
> 2 cycles	58 (41.73)	31 (39.74)	

*IC* induction chemotherapy, *ICRT* induction chemotherapy plus radiotherapy, *ICCCRT* concurrent chemotherapy plus radiotherapy, *TP* docetaxel/paclitaxel plus cisplatin, *FP* cisplatin plus fluorouracil

All patients completed the full course of VMAT. For IC treatment, 45 patients completed one cycle, 83 patients completed two cycles and 89 patients completed three or more cycles; 155 patients received the TP regimen and 29 patients received the FP regimen. Two patients did not complete the course of CC due to severe toxicities in the IC/CCRT arm.

### Response and survival outcomes

**Fig. 2:**
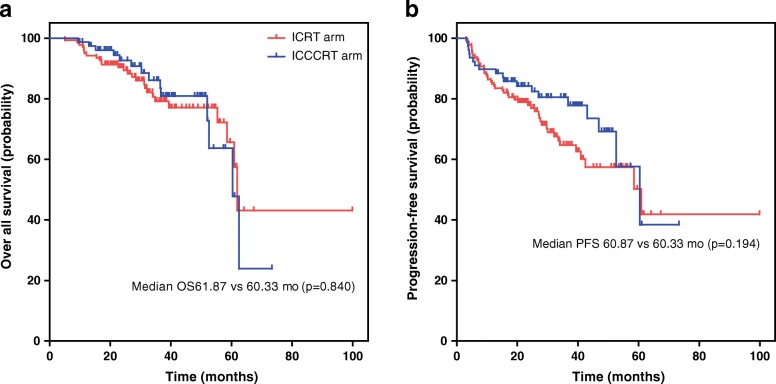
Kaplan-Meier survival curves for stage II-IVB NPC patients in the ICRT and ICCCRT arms. **a** overall survival **b** progression-free survival

**Fig. 3:**
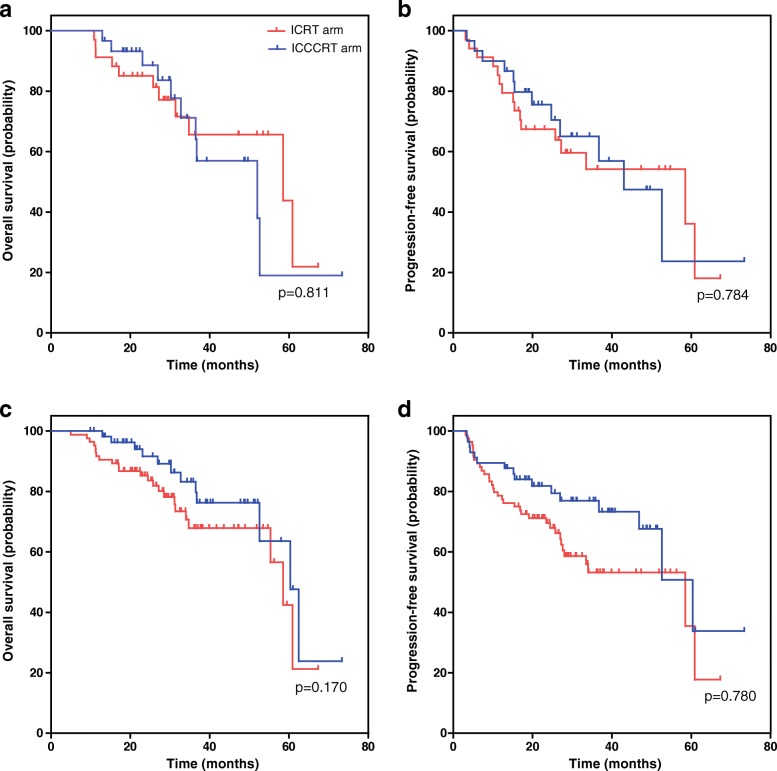
Kaplan-Meier survival curves for subgroup patients with NPC in the ICRT and ICCCRT arms. **a** overall survival for NPC with T3–4 category **b** progression-free survival for NPC with T3–4 category. **c** overall survival for NPC with N2–3 category **d** progression-free survival for NPC with N2–3 category

Treatment response was assessed for all patients 3 months after radiotherapy. The disease control rate (DCR) and objective response rate (ORR) for IC/RT and IC/CCRT arms were 91.37% vs. 93.60% (*p* = 0.56), and 61.87% vs. 66.67% (*p* = 0.46), respectively. The median follow-up of all patients was 62 months. There was no significant difference in median OS (61.87 vs. 60.33 months; *p* = 0.84) and median PFS (60.87 vs. 60.33 months; *p* = 0.19) between IC/RT and IC/CCRT arms. Up to the date of final analysis, 27 people died in the IC/RT arm, and 14 died in the IC/CCRT arm. The 3-year OS, PFS rates were 79.1%, 64.7% for the IC/RT arm, and 86.1%, 80.5% for the IC/CCRT arm, respectively. The 5-year OS, PFS rates were 57.5%, 41.8% for the IC/RT arm, and 47.8%, 38.4% for the IC/CCRT arm, respectively. There was no significant difference in survival between the two groups as shown in Table 2 and Fig. 2. There was also no significant difference in subgroup survival analysis according to stage T3–4 category and N2–3 category (*p* > 0.05, Fig. 3).

**Table 2:** Comparison of the survival rates for ICRT VS ICCCRT

Variable	ICRT arm	ICCCRT arm
OS (%)		
At 3-years	79.1	86.1
At 5-years	57.5	47.8
PFS (%)		
At 3-years	64.7	80.5
At 5-years	41.8	38.4

*ICRT* induction chemotherapy plus radiotherapy, *ICCCRT* concurrent chemotherapy plus radiotherapy

### Prognostic factors

Table 3 shows the results of univariate and multivariate analysis with the Cox proportional hazards model. It shows that age and N category were significant prognostic factors for both OS and PFS, and T category was a significant prognostic factor for OS. Treatment modality (IC/RT vs. IC/CCRT) was not correlated with survival for either OS or PFS.

**Table 3:** Univariate and multivariate analysis of the associations between factors affecting OS and PFS in patients

Outcome	Univariate	Multivariate	
	*P*-value	Hazard ratio (95% CI)	*P*-value
Overall survival			
Sex female vs. male	0.24	0.57 (0.22–1.47)	0.24
Age ≤ 54 years vs. > 54 years	0.003^a^	0.43 (0.22–0.82)	0.01^a^
T category T1–2 vs.T3–4	0.001^a^	0.52 (0.27–0.99)	0.046^a^
N category N0-1vs. N2–3	0.001^a^	0.34 (0.13–0.85)	0.02^a^
Treatment arm ICRT vs. IC/CCRT	0.84	1.35 (0.70–2.61)	0.37
Progression-free survival			
Sex female vs. male	0.49	0.74 (0.39–1.39)	0.34
Age ≤ 54 years vs. > 54 years	< 0.001^a^	0.36 (0.21–0.60)	< 0.001^a^
T category T1–2 vs.T3–4	0.01^a^	0.67 (0.40–1.14)	0.14
N category N0-1vs. N2–3	0.003^a^	0.49 (0.26–0.93)	0.03^a^
Treatment arm ICRT vs. IC/CCRT	0.19	1.62 (0.94–2.79)	0.08

*ICRT* induction chemotherapy plus radiotherapy, *ICCCRT* concurrent chemotherapy plus radiotherapy, *CI* confidence interval

^a^statistical significant

### Toxicities

The most common toxicities related to treatment were listed in Table 4. There were no treatment-related deaths and grade 3–4 of kidney disfunction toxicities observed in our study. There were no significant differences of acute hematological toxicities and grade 3–4 non hematological adverse events between the two groups (*p* > 0.05) during the course of IC.

**Table 4:** Profile of treatment-related toxicities

	ICRT arm (*n* = 139)	IC/CCRT arm (*n* = 78)	*P*-value
Grade 3–4 adverse events during IC, n (%)			
Hematological			
Leukopenia/neutropenia	26 (18.71)	20 (25.64)	0.23
Thrombocytopenia	1 (0.72)	0 (0)	1.00
Anemia	2 (1.44)	0 (0)	0.54
Non-Hematological			
Nausea/vomiting/diarrhea	23 (16.55)	12 (15.38)	0.82
Liver disfunction	0 (0)	2 (2.56)	0.13
Kidney disfunction	0 (0)	0 (0)	NA
Grade 3–4 adverse events during RT, n (%)			
Hematological			
Leukopenia/neutropenia	5 (3.60)	16 (20.51)	< 0.001^a^
Thrombocytopenia	2 (1.44)	4 (5.13)	0.19
Anemia	2 (1.44)	2 (2.56)	0.30
Non-Hematological			
Nausea/vomiting/diarrhea	3 (2.16)	32 (41.03)	< 0.001^a^
Skin reaction	58 (41.73)	39 (50.00)	0.24
Mucositis	41 (29.50)	37 (47.44)	0.01^a^
Grade 3–4 late toxicities			
Xerostomia	48 (34.53)	38 (48.72)	0.04^a^
Ear (deafness/otitis)	39 (28.06)	27 (34.62)	0.31
Cranial neuropathy	9 (6.47)	4 (5.13)	0.69
Neck tissue damage	28 (20.14)	24 (30.77)	0.08

*IC* induction chemotherapy, *RT* radiotherapy, *ICRT* induction chemotherapy plus radiotherapy, *ICCCRT* concurrent chemotherapy plus radiotherapy, *NA* none available

^a^statistical significant

The IC/CCRT arm showed significantly higher rates of grade 3–4 leukopenia/neutropenia (3.60% vs. 20.51%, *p* < 0.001), gastrointestinal disorder (nausea/vomiting/diarrhea, 2.16% vs. 41.03%, *p* < 0.001), mucositis (29.50% vs. 47.44%, *p* = 0.01), and xerostomia (34.53% vs. 48.72%, *p* = 0.04) compared with the IC/RT arm. No significant differences in thrombocytopenia, anemia, skin reaction ear problems (deafness/otitis), cranial neuropathy, and neck tissue damage were found between the two arms (*p* > 0.05).

## Discussion

In this study, the survival outcomes and toxicities between IC followed by VMAT alone and IC plus CCRT in the treatment of stage II-IVB NPC patients were compared. There was no significant difference in OS and PFS observed for the addition of CC to VMAT after IC compared with IC plus VMAT alone. However, the risk of adverse effects, such as leukopenia/neutropenia, gastrointestinal events, mucositis and xerostomia were increased with the addition of CC.

The efficacy of chemotherapy plus conventional radiotherapy has been confirmed in many clinical researches [8, 20–22], but the best sequence of chemotherapy with RT has not been well concluded and the value of CC added to VMAT after IC remains unknown. IC followed by RT alone had been reported to achieve benefit in response rate (RR) [15], DFS [14], reduce regional recurrence and distant metastasis [3, 4, 23]. In this study, IC plus VMAT alone also provided satisfactory outcomes with a DCR of 91.37% and an ORR of 61.87% at 3 months after RT.

The benefit of IC/CCRT in the treatment of NPC is still controversial. Sun et al. [4] demonstrated that IC/CCRT significantly improved OS, FFS rates of NPC patients. Tan et al. [24] also reported a 3-year OS rate of 94.3% in NPC with IC/CCRT. However, several trials failed to achieve superior survival outcomes by adding CC with RT after IC compared with IC/RT alone. Lin et al. [23] stated that additional CC to IC/RT offered no significant advantage for further improvement of local and regional control. Huang et al. [9] reached a similar conclusion in their study. In the trial conducted by Su et al. [25], NPC patients had similar OS, MFS, and DFS when treated with RT-based modalities, including IC plus RT, IC plus CCRT. In this study, VMAT was applied as the RT method in evaluation the efficacy and toxicities of IC/RT and IC/CCRT in the treatment of NPC. We also failed to observe a better outcome for IC/CCRT with VMAT in terms of either OS (*p* = 0.84) or PFS (*p* = 0.19) compared with IC plus VMAT alone. Multivariate analysis also indicated that CC was not an independent prognostic factor for either OS or PFS.

It is believed that local recurrence and distant metastasis are the major causes of failure in the treatment of NPC patients [26–29]. The ability of CC to control distant metastasis was relatively limited according to many studies [30–32]. Compared with CC, IC is capable of delivering chemotherapy drugs through the vasculature and eradicating micrometastases more effectively by administering the drugs before radiotherapy. It is expected to regress tumor extension, increase tumor radio-sensitivity, protect normal tissue at risk, prevent tumor progression due to the long waiting time before RT, and finally improve the local and distant control [33, 34]. On the other hand, IC is more likely to be tolerated by patients in the initial stage of treatment [35], thus increases their compliance [36]. More importantly, the shrunk primary lesions after IC can provide a wider boundary for RT, which is particularly important for NPC patients with tumor invasion or in close proximity to many critical normal tissues [37–39]. In this study, promising results on 3-year OS, PFS rates (79.1%, 64.7%) and median OS and PFS (61.87 and 60.87 months) were achieved with IC/RT for NPC patients.

IMRT is able to escalate the dose to the target while sparing the adjacent critical structures [40, 41], and has shown remarkable benefits in local control and relapse-free survival of NPC patients [31, 42, 43]. IMRT/VMAT has been widely applied clinically in the treatment of NPC. Lin et al. [23] reported that there were no significant differences in distant disease-free survival (DDFS), DFS, OS (84.5% vs. 82.6% vs. 89.1% and 85.8% vs. 80.3%, 89.2%) between IMRT alone and IMRT plus CC group (*p* > 0.05).

Drug-related adverse effects of CC lead to the interruption of treatment in many NPC patients [22, 44, 45]. In the INT-0099 trial [8], the proportion of patients who completed the scheduled CC was only 63% due to excess toxicities. Lin et al. [23] and Sun et al. [28] reported that the total occurrence rates of grade 3–4 acute toxicities in patients receiving CC were higher than those receiving RT alone. Our study also demonstrated that additional CC increased the occurrence rates of grade 3–4 toxicities, especially leukopenia/neutropenia (*p* < 0.001), gastrointestinal events (nausea/vomiting/diarrhea) (*p* < 0.001), mucositis (*p* = 0.01) and xerostomia (*p* = 0.04). These serious toxicities could obviously reduce the compliance of patients during CCRT.

One limitation of current study is that this is a retrospective methodology from a single-institution experience. The impact of various treatments related outcomes could not be fully evaluated. The number of patients enrolled may not be sufficient enough and the follow-up duration of the study may not be long enough. External validation using other large database for further evaluating the role of CC for NPC.

## Conclusion

In summary, there was no significant benefits on survival observed with IC/CCRT compared with IC/RT alone in the treatment of stage II-IVB NPC patients. On the contrary, more severe side effects were associated with IC/CCRT. CC should be used with caution in the treatment of NPC combined with IC and VMAT.

## Abbreviations

2D-CRT: Two-dimensional conventional radiotherapy
CCRT: Concurrent chemotherapy
CT: Computed tomography
CTCAE: Common Terminology Criteria for Adverse Events
CTV: The first clinical tumor volume
CTV2: The second CTV
DCR: Disease control rate
DFS: Disease-free survival
FP: Cisplatin plus fluorouracil
GTV: Gross tumor volume
IC: Induction chemotherapy
IMRT: Intensity-modulated radiotherapy
MRI: Magnetic resonance imaging
NPC: Nasopharyngeal carcinoma
OS: Overall survival
PFS: Progression-free survival
PTV: Planning target volume
RECIST: Response evaluation criteria in solid tumors
RR: Response rates
RT: Radiotherapy
SIB: Simultaneous integrated boost
TP: Docetaxel/paclitaxel plus cisplatin
TPS: Treatment planning system
